# New Work on Medicine

**Published:** 1848-12

**Authors:** 


*New Work on Medicine.*—The Southern Medical and Surgical Journal announces the publication of a new work on medicine, by Dr. Tomlinson Fort, of Milledgeville, Georgia. The editor states that Dr. Fort has, for forty years, occupied the most prominent position of any man in the medical profession of Georgia.

